# Targeted APC Activation in Cancer Immunotherapy to Enhance the Abscopal Effect

**DOI:** 10.3389/fimmu.2019.00604

**Published:** 2019-04-02

**Authors:** Nathan Suek, Luis Felipe Campesato, Taha Merghoub, Danny N. Khalil

**Affiliations:** ^1^Swim Across America and Ludwig Collaborative Laboratory, Immunology Program, Parker Institute for Cancer Immunotherapy, Memorial Sloan Kettering Cancer Center, New York, NY, United States; ^2^Department of Medicine, Weill Cornell Medical College, New York, NY, United States

**Keywords:** abscopal effect, APC activation, DC, immunogenic cell death (ICD), CD40L, TLR9

## Abstract

In oncology, the “abscopal effect” refers to the therapeutic effect on a distant tumor resulting from the treatment of local tumor (e. g., ablation, injection, or radiation). Typically associated with radiation, the abscopal effect is thought to be mediated by a systemic antitumor immune response that is induced by two concurrent changes at the treated tumor: (1) the release of tumor antigens and (2) the exposure of damage-associated molecular patterns. Therapies that produce these changes are associated with immunogenic cell death (ICD). Some interventions have been shown to cause an abscopal effect without inducing the release of tumor antigens, suggesting that release of tumor antigens at baseline plays a significant role in mediating the abscopal effect. With tumor antigens already present, therapies that target activation of APCs alone may be sufficient to enhance the abscopal effect. Here, we discuss two therapies targeted at APC activation, TLR9 and CD40 agonists, and their use in the clinic to enhance the abscopal effect.

## Introduction

The abscopal effect (derived from the Latin “ab” meaning away from and “scopus” meaning target) refers to the local destruction of a tumor which results also in the regression of a distant tumor. The phenomenon has been well-described in pre-clinical models, often in the context of radiation. For example, when combined with Flt3-L, irradiation not only resulted in control of the primary tumor but also of a non-irradiated secondary tumor (1). Though described in pre-clinical settings, the abscopal effect is still relatively rare in patients. In one study of 34 patients with metastatic prostate cancer, treatment with radiation and the immunotherapy, ipilimumab, resulted in a complete response at local and distant tumors for one patient (3%) (2). Ionizing radiation (IR) is thought to induce the abscopal effect via two changes at the treated tumor: (1) release of tumor associated antigens (TAAs) and (2) release of damage associated molecular patterns (DAMPs) which activate antigen presenting cells (APCs). We will examine the relative contribution of both factors to the abscopal effect and the role of IR in inducing each.

## Release of Antigens and Exposure of DAMPs

IR damages a tumor cell's DNA which can result in ICD. In ICD, the tumor cell releases antigen and enhances phagocytosis by APCs through signals like calreticulin, which facilitates phagocytosis, and ATP, which attracts APCs (3). The net result is the presentation of tumor antigens by APCs. While tumor cells have been reported to act as antigen presenting cells, we will use the term APC to refer to professional APCs such as dendritic cells (DC) and macrophages. However, antigen presentation by immature DCs can lead to T cell tolerance as T cells become anergic, suppressive, or are simply deleted (4). To mediate the abscopal effect, radiation is thought not only to release tumor antigens but also DAMPs that activate APCs. DAMPs include HMGB1, ATP, and non-nuclear DNA (3). While DAMPs exert their effects by various mechanisms, they converge on the same functional outcome: activation of APCs that can initiate an adaptive immune response.

**Figure 1:**
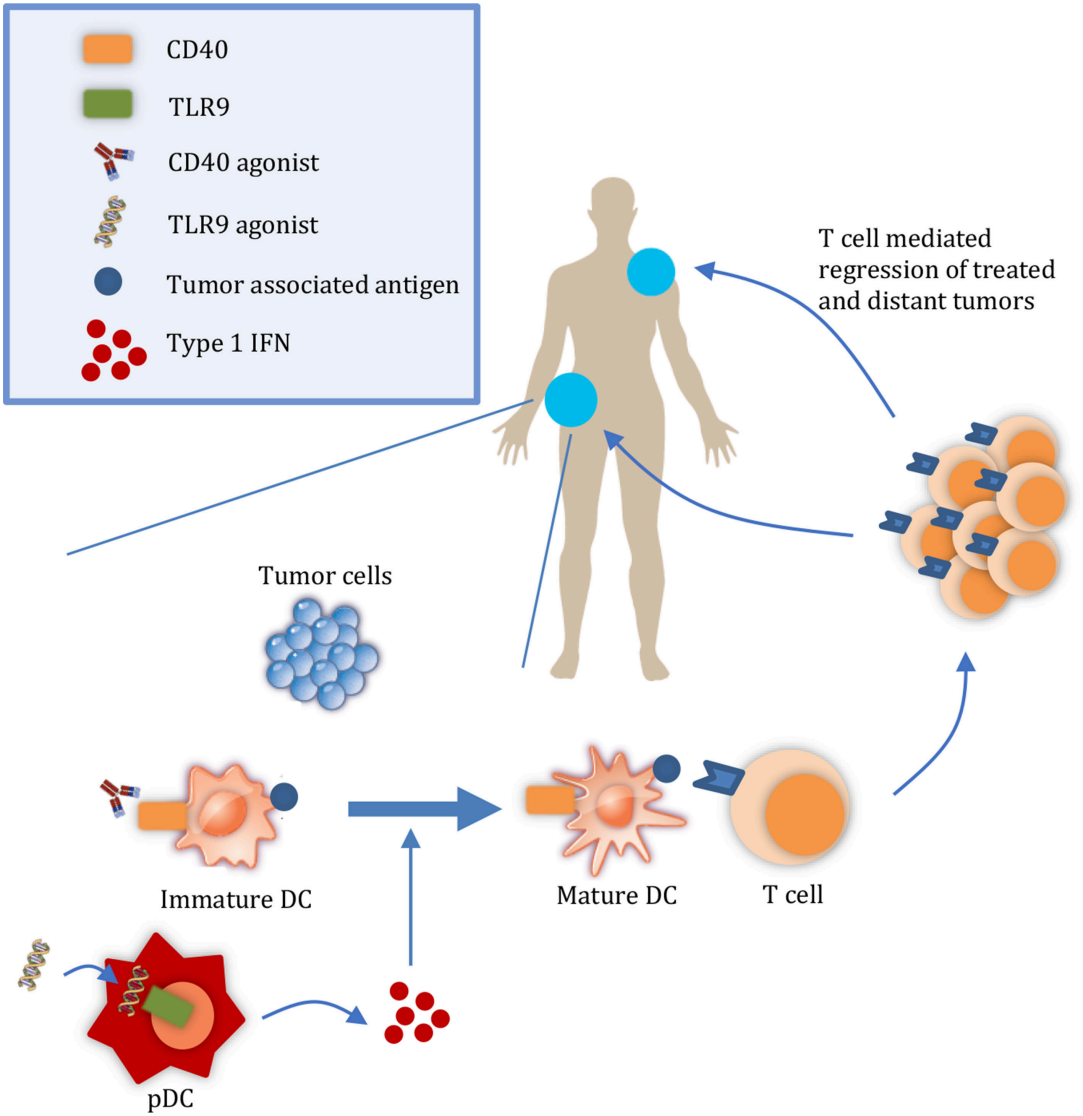
Targeted therapies that promote APC activation enhance the abscopal effect. At a local tumor, administration of therapies such as CD40 and TLR9 agonists results in the maturation of DCs. Mature DCs can then prime T cells to regress both the local tumor and also distant tumors. DC, dendritic cell; pDC, plasmacyotid dendritic cell.

It is important to consider the relative contributions of both the release of tumor antigens and of DAMPs to the abscopal effect. During tumor growth, antigens from malignant cells undergoing chronic turnover are engulfed by DCs. However, DAMPs may not be released in sufficient quantities to consistently mature them (5, 6). For example, in a study by Vicari et al. tumor infiltrating dendritic cells (TIDCs) at baseline were shown to present antigen albeit in an immature state. Upon treatment with CpG and IL10R blocking antibody to activate TIDCs, tumors were regressed by an adaptive immune response (6). Given the presence of tumor antigens in the microenvironment, interventions that activate APCs without releasing additional antigens may be sufficient to initiate a systemic immune response and abscopal effect. Considering the rarity of the abscopal effect with radiation, other therapies that promote DC activation in a targeted manner may increase its frequency (7) (Figure 1). Here, we discuss TLR9 and CD40 as promising therapeutic targets to enhance DC activation and summarize their progress in clinical development.

## TLR9

Modern cancer immunotherapy arguably began with Dr. William Coley's intratumoral injections of bacterial lysates, derisively called “Coley's toxin.” It was later determined that bacterial DNA within these lysates, specifically the CpG sequence motif, was the component responsible for eliciting an immune response (8). Unmethylated cytosine-phosphate-guanine (CpG) binds to TLR9 which, in humans, is expressed primarily by (1) B cells and (2) plasmacytoid dendritic cells (pDCs) (9). TLR9 signals through each cell type to initiate a differing cascade of immune effects. TLR9 activation on B cells enhances their differentiation into antibody-secreting plasma cells (10). TLR9 activation on pDCs results in several effects: (1) secretion of type-1 interferons (4) (2) secretion of Th1 type cytokines (e.g., TNFα, IFNγ, IL2) (10) (3) expression of TNF-related apoptosis-inducing ligand (TRAIL) which can induce tumor cell death directly (9) and (4) expression of co-stimulatory molecules (e.g., CD80, CD86) and lymph node homing signal CCR7 (9). While the APC function of pDCs is debated (11), pDC secretion of type-1 interferons, primarily IFNα, is thought to be the dominant effect by which TLR9 signaling induces antitumor immunity (10). IFNα has direct effects on tumors including the inhibition of angiogenesis (12), antiproliferative effects (13), as well as increased MHC I expression and thus enhanced immunogenicity (13). Its effect on immune cells include the enhanced ability of NK cells to kill and produce IFNγ as well as the maturation of conventional DCs (13). The ability to mature DCs is particularly appealing as a therapy to enhance the abscopal effect.

Interest in the clinical use of TLR9 agonists has waxed and waned throughout the years. In mouse studies, therapies involving CpG have induced extremely potent abscopal responses, often resulting in complete regression of treated tumors as well as distant, non-treated tumors (6, 14–16). Early human data was also promising. As monotherapy, TLR9 agonists have been shown in several phase I and II to result in objective responses, and even some complete responses, in cutaneous T cell lymphoma (17), basal cell carcinoma (18), and melanoma (18). However, in two phase III trials for advanced NSCLC, TLR9 in combination with either chemotherapy regimens paclitaxel/carboplatin (19) or gemcitabine/cisplatin (20) did not extend overall survival compared to chemotherapy alone. In fact, addition of TLR9 resulted in increased adverse effects which led to early discontinuation of TLR9 administration in those studies. Interest in TLR9 faltered after these trials and efforts were scaled back on CpG agents like PF-3512676 (10). The discrepancy between the promising preclinical data and disappointing clinical results may be partially attributed to the broad expression of TLR9 in mice (in nearly all myeloid cells) compared to narrow expression in humans (primarily B cells and pDCs) (8).

With the recent successes of immune checkpoint blockade, there has been renewed interest in TLR9 agonists for their potential in combination therapies with T cell activating agents. Various combinations are currently being tested in clinical trials and are detailed in Table 1. These trials improve upon past trials of such agents for at least two reasons (21). First, whereas previous failed trials (19, 20) used subcutaneous systemic administration, current trials often focus on intratumoral injection (e.g., NCT03410901, NCT03445533). Intratumoral injection is thought to increase potency while also avoiding systemic toxicity. Indeed, one CpG agent has been shown to regress tumors when given intratumorally (7) whereas the same drug had little to no effect when given systemically (22). Second, the impact of TLR9 agonists in the past may have been curtailed by negative feedback mechanisms such as increased PD-1 signaling. Ongoing trials combining TLR9 with ipilimumab (8) and pembrolizumab (23) attempt to address these barriers. Recent trials (23, 24) employing these strategies have been well-tolerated while regressing local, treated tumors, and untreated, abscopal tumors. Such studies merit further investigation to further elucidate the effectiveness of CpG for inducing systemic immunity.

**Table 1:** Selected TLR9 agonists in clinical development.

**Drug**	**Developer**	**Conditions**	**Combination**	**Clinical trials**
SD-101	Dynavax	B cell Hodgkin's Lymphoma B cell Lymphoma Melanoma Head and neck squamous cell carcinoma Lymphoma	SD-101 Anti-OX40 Ab (BMS 986178) Radiation SD-101 Ipilimumab Radiation SD-101 Pembrolizumab SD-101 Epacadostat Radiation	NCT03410901 NCT02254772 NCT02521870 NCT03322384
IMO-2125	Idera	Melanoma Melanoma	IMO-2125 Ipilimumab IMO-2125 Ipilimumab/Pembrolizumab	NCT03445533 NCT02644967
CMP-001	Checkmate Pharmaceuticals	Melanoma Melanoma NSCLC	CMP-001 Pembrolizumab CMP-001 Nivolumab CMP-001 Atezolizumab Radiation	NCT03084640 NCT03618641 NCT03438318
MGN1703	Mologen	Advanced solid cancers	MGN1703 Ipilimumab	NCT02668770

*A selection of clinical trials of interest are shown. Information compiled from clinicaltrials.gov*.

## CD40

The TNF superfamily receptor CD40 is expressed on hematopoietic cells such as DCs, B cells, monocytes, and macrophages, non-hematopoietic cells such as epithelial cells and fibroblasts, as well as tumor cells in melanoma and lung cancer (25). Its ligand, CD40L, is expressed by CD4 T cells. Ligation of CD40 results in activation of the cell on which is expressed (26). On B cells, CD40 signaling results in class switching, somatic hypermutation, formation of long lived plasma, and memory cells (25), and enhanced antigen presenting function (27). On DCs, CD40 signaling results in upregulation of costimulatory molecules (e.g., CD80, CD86), production of cytokines (e.g., IL-12) (26), enhanced expression and stability of MHC, and increased expression of factors which promote survival (e.g., Bcl-XL) (25). CD40 signaling occurs through two categories of adapter protein: (1) TNFR-associated factors (TRAFs) and (2) Jak family kinase 3 (JAK3). This leads to activation of various signaling pathways including MAPK, PI3K, PLCγ, and NF-kβ. Details of these signaling pathways have been described elsewhere (25).

In preclinical models, agonist CD40 antibodies have been shown to be effective at regressing tumors (28, 29). The mechanism of agonist CD40 antibodies can be subdivided into T cell independent and dependent effects. The T cell independent effects include direct apoptotic signaling on CD40+ tumors (30), targeting of CD40+ tumors for ADCC or complement-dependent cytotoxicity (CDC), and activation of other effector cells including NK cells (31) and macrophages (32) to regress tumors. The T cell dependent effects are mediated by activation of APCs which allow them to prime tumor specific CD8 T cells. In treatment with anti-CD40 mAb, depletion of CD4 T cells does not affect efficacy, suggesting that the CD40 mAb replaces the need for CD40L from helper T cells (33).

While no anti-CD40 antibodies have been approved by the FDA, several are in active clinical development and detailed in Table 2. In the clinic, CD40 agonists have had moderate therapeutic activity. CD40 agonist, CP-870,893 as single agent has resulted in 14% objective response rate in a study of advanced solid cancers (34). Of note, one of these patients with melanoma went on to have a complete response that has lasted over a decade (26). However, in another study of advanced solid cancers, single agent CP-870,893 resulted in no clinical benefit (35). In combination with chemotherapy, CP-870,893 has had a 20% response in various advanced solid tumors (36). In the case of metastatic pancreatic cancer, this was higher than response rates with chemotherapy alone (37). Other CD40 agonists such as Chi Lob 7/4 have demonstrated no objective responses in initial clinical studies (27). In terms of toxicity, CD40 agonists have been associated with important adverse effects. For example, CP-870,893 resulted in CRS in a majority of patients and has been the dose limiting toxicity (38). These two issues, moderate efficacy, and toxicity, have hindered the clinical development of CD40 agonists.

**Table 2:** Selected CD40 agonists in clinical development.

**Drug**	**Developer**	**Fc**	**Conditions**	**Combination**	**Clinical Trials**
CP-870,893	Pfizer/VLST	IgG2	Advanced solid tumors Pancreatic adenocarcinoma Metastatic solid tumors	CP-980,893 CP-980,893 Gemcitabine CP-980,893 Paclitaxel Carboplatin	NCT01103635 NCT01456585 NCT00607048
Dacetuzumab (SGN-40)	Seattle Genetics	IgG1	Lymphoma	SGN-40	NCT00435916
Chi Lob 7/4	University of Southampton	IgG1	Advanced malignancies	Chi Lob 7/4	NCT01561911
APX005M	Apexigen	IgG1	Melanoma Pancreatic adenocarcinoma	APX005M Pembrolizumab Gemcitabine Nab-Paclitaxel APX005M Nivolumab	NCT02706353 NCT03214250

*A selection of clinical trials of interest are shown. Information compiled from clinicaltrials.gov*.

To address efficacy, it is important to consider the mechanism by which these antibodies induce agonism. CD40 mAbs require crosslinking (i.e., oligomerization) of the CD40 receptor to induce the agonistic effect. Crosslinking can be enhanced by an *in trans* interactions between the CD40 mAb Fc region and an Fc receptor (FcR) expressed on a neighboring cell (39). As such, some have worked to improve CD40 agonist activity through Fc engineering to enhance the Fc-FcR interaction (27, 40–43). Others have suggested that the mAb formatted as an IgG2b has a compact hinge structure which may mediate effective crosslinking in the absence of the Fc receptor (44). Current CD40 agonists do not achieve optimal efficacy for several different reasons. Most CD40 agonists have been IgG1 and this human isotype has been shown to bind poorly to FcγRIIb (45), which is thought to be the main Fc receptor that mediates effective crosslinking (33). IgG2 mAb in development include CP-870,893 which shuffles between the IgG2a and IgG2b formats thereby limiting its potency (44). For these reasons, many CD40 agonists, both IgG1 and IgG2, likely fail to achieve their full therapeutic efficacy due to suboptimal crosslinking. Higher order oligomerization of the CD40 receptor (i.e., crosslinking) is thought to result in greater activation of downstream pathways such as NF-kβ (46) and drive greater immunostimulation (25). In a preclinical study of recombinant CD40L, forming CD40L into higher order oligomers significantly increased B cell activation (47). Future development of CD40 agonists should focus on achieving efficient clustering to maximize therapeutic efficacy.

To address toxicity, researchers have investigated local injections of CD40 agonists to avoid systemic adverse effects. For example, at the same dose, local injection was superior to systemic administration of CD40 agonists (48) while also reducing biodistribution of the antibody in the liver and possibly hepatotoxicity (38). This superior efficacy of local administration along with decreased toxicity has been demonstrated in multiple tumor models (41, 49–51). Efficacy and toxicity go hand in hand. Increased dosing could be one strategy to compensate for suboptimal efficacy, but in the case of CD40 agonists, this is problematic due to issues with toxicity. Until optimal dosing and route of administration is established, it is unlikely that CD40 agonists will have reached their maximum therapeutic potential (26).

## APC Activation as Part of Combination Immunotherapies

As an immunotherapeutic strategy, focusing on antigen presentation represents only one step in mediating a systemic antitumor response. Other aspects—from T cell infiltration to immunosuppression by myeloid cells—remain critical steps for T-cell mediated tumor control. For example, in one study, the efficacy of checkpoint blockade was significant enhanced by combination with PI3k-γ, which switches tumor associated myeloid cells from an immunosuppressive to immunostimulatory phenotype (52). Therefore, combinatorial approaches that target multiple aspects of the cycle are promising strategies for treatment. Future studies that target several aspects of antitumor immunity, including APC activation, are likely to improve patient outcomes in the years ahead.

## Author Contributions

All authors researched data for article, contributed to discussion of the content, wrote the manuscript and reviewed/edited the article before submission.

### Conflict of Interest Statement

TM is a consultant for Immunos Therapeutics and Pfizer. TM is a co-founder with equity in IMVAQ therapeutics. TM receives research funding from Bristol-Myers Squibb, Surface Oncology, Kyn Therapeutics, Infinity Pharmaceuticals Inc., Peregrine Pharmeceuticals Inc., Adaptive Biotechnologies, Leap Therapeutics Inc., and Aprea. TM is an inventor on patent applications related to work on Oncolytic Viral therapy, Alpha Virus Based Vaccine, Neo Antigen Modeling, CD40, GITR, OX40, PD-1 and CTLA-4. DK is an inventor on patent applications related to CD40. LC is a consultant for Merck. The remaining author declares that the research was conducted in the absence of any commercial or financial relationships that could be construed as a potential conflict of interest.
